# Brain pathology in relation to somatic diseases: Exploring the body–brain crosstalk

**DOI:** 10.1111/joim.70119

**Published:** 2026-06-02

**Authors:** G. Grande, M. Valletta, F. Gasparini, D. L. Vetrano, M. Canevelli, J. R. Bacci, C. Qiu, A. Marengoni, M. Toccaceli Blasi, H. Xu, L. Fratiglioni, M. M. Mielke

**Affiliations:** ^1^ Aging Research Center, Department of Neurobiology, Care Sciences and Society Karolinska Institutet and Stockholm University Stockholm Sweden; ^2^ Stockholm Gerontology Research Center Stockholm Sweden; ^3^ Department of Human Neuroscience Sapienza University of Rome Rome Italy; ^4^ Department of Epidemiology and Prevention Wake Forest University School of Medicine Winston‐Salem North Carolina USA; ^5^ Department of Clinical and Experimental Sciences University of Brescia Brescia Italy; ^6^ Barcelonaβeta Brain Research Center (BBRC) Pasqual Maragall Foundation Barcelona Spain; ^7^ Division of Clinical Geriatrics, Department of Neurobiology, Care Sciences and Society Karolinska Institutet Stockholm Sweden

**Keywords:** Alzheimer's disease, body–brain crosstalk, brain pathology, co‐pathology, somatic diseases

## Abstract

Several somatic diseases have been consistently linked with an increased dementia risk. However, the underlying neuropathological substrates remain poorly characterized. This narrative review aims to summarize evidence on the association between common age‐related somatic conditions (i.e., heart diseases, type 2 diabetes, kidney disease, liver diseases, lung disease, and anemia) and neuropathological findings, encompassing both Alzheimer's disease (AD)‐related pathology (amyloid and tau deposition) and non‐AD co‐pathologies (i.e., neuronal loss, neuroinflammation, cerebrovascular lesions, and non‐AD proteinopathies). We conducted a PubMed search for human studies examining these associations using postmortem data, brain imaging, or cerebrospinal fluid biomarkers. Evidence was qualitatively synthesized and graded according to its strength. Strong and consistent associations were observed between most examined somatic diseases and global neuronal loss or brain atrophy, as well as cerebrovascular lesions. In contrast, evidence associating these conditions with amyloid and tau pathology was limited and inconsistent. No studies systematically examined neuroinflammation or non‐AD proteinopathies in relation to somatic diseases. Together, the available evidence suggests that somatic diseases are unlikely to primarily drive AD pathology but instead contribute to brain damage through a combination of non‐AD processes, particularly cerebrovascular injury and diffuse neuronal loss. These findings highlight the need to increase our knowledge of “mixed dementia” and move beyond a purely brain‐centric view of dementia, especially in older adults with complex clinical profiles.

## Graphical abstract

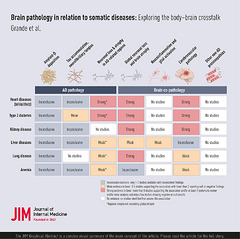

## Introduction

Dementia is a clinically heterogeneous syndrome caused by multiple biologically complex mechanisms. The neuropathological hallmarks of Alzheimer's disease (AD), that is, the accumulation of amyloid‐β (Aβ) plaques and neurofibrillary tau tangles, represent the most common neuropathological substrate of dementia [1, 2]. However, AD‐related pathology rarely occurs in isolation. In older adults, mixed pathology—namely, the coexistence of AD‐related alterations with vascular lesions, neuroinflammation, and other proteinopathies—is almost invariably observed [3, 4, 5].

Systemic health is essential for maintaining brain health, and numerous somatic conditions have been associated with the development of dementia [6, 7, 8, 9], as well as with prodromal stages of the disease [10]. Cardiovascular [11] and metabolic disorders [12, 13, 14] are clear examples of this well‐established body–brain crosstalk. For instance, consistent evidence supports an association between heart diseases, particularly atrial fibrillation [15] and heart failure [16], and a faster progression to mild cognitive impairment and clinical dementia [11]. Moreover, these conditions are associated with a poorer prognosis, as affected individuals tend to have shorter survival following a dementia diagnosis.

The biological mechanisms underlying these associations may involve multiple processes [6], including vascular dysfunction (e.g., atrial fibrillation [17]), systemic inflammation [11], and chronic hypoxia (e.g., pulmonary disease [18]), all of which can contribute to neuronal damage and cognitive decline through pathways that are mostly independent of classical AD pathology [19]. Importantly, some of these conditions have also been hypothesized to accelerate AD‐like processes, including Aβ accumulation [20] and other cardinal neuropathological changes [21]. For instance, coronary artery disease has been associated with greater plaque and tangle burden in an autopsy study [21], and broader evidence supports a connection between Aβ deposition and vascular pathologies [22, 23].

Nonetheless, although many of these somatic diseases are common in older adults and have been associated with the clinical expression of dementia, their relationship with underlying neuropathological substrates remains less clearly defined. In particular, it is unclear whether these conditions primarily contribute to AD‐related pathology, to non‐AD brain changes, or to a combination of both. Clarifying the association of somatic diseases with neuropathological changes may advance our understanding of dementia pathophysiology, facilitate the development of preventive and therapeutic strategies, and improve individuals’ stratification and prognostication.

This narrative review aims to qualitatively synthesize and appraise the available evidence on the association between common age‐related somatic diseases and neuropathological substrates of dementia, including both AD‐related pathology and non‐AD co‐pathologies, such as vascular injury, neuroinflammation, and non‐AD proteinopathies. By focusing on brain pathology from human studies with postmortem and biomarker‐based endpoints, we seek to clarify how systemic health may contribute to neurobiological processes underlying dementia development in older adults.

## Methods

We conducted a narrative review to examine the association between pre‐specified somatic diseases and neuropathological substrates. We focused on somatic conditions commonly affecting older adults, selected based on two criteria: (1) high prevalence in late life, and (2) consistent prior evidence of association with cognitive decline and/or a clinical diagnosis of dementia. The somatic diseases included were heart diseases (including heart failure, atrial fibrillation, and ischemic heart disease), type 2 diabetes, kidney disease, liver diseases, lung disease, and anemia. We examined a broad range of neuropathological outcomes, encompassing Aβ deposition and neurofibrillary tangles, as well as non‐AD co‐pathologies, such as neuronal loss, neuroinflammation or altered microglial phenotypes, cerebrovascular lesions, and other proteinopathies (e.g., α‐synuclein and transactive response DNA binding protein of 43 kDa [TDP‐43] aggregates). These processes were included because they frequently co‐occur with AD‐related pathology and contribute to the clinical expression of dementia [24, 25, 26, 27].

Targeted literature searches were performed in the PubMed database using combinations of MeSH terms and free‐text keywords for each chronic disease (e.g., “Kidney Diseases” [Mesh], “Chronic Kidney Disease,” and “CKD”) and for neuropathological features (e.g., “Cerebrovascular Lesions,” “White Matter Lesions,” “TDP‐43,” “Neuroinflammation,” “Lewy Bodies,” and “Proteinopathies”). We included studies conducted in humans and published in English. Eligible studies were postmortem/autopsy investigations and those using biomarker‐based endpoints, including neuroimaging (amyloid PET, tau PET, FDG‐PET, and MRI) and cerebrospinal fluid (CSF) biomarkers.

Given the wide range of topics addressed, this review was not intended to be systematic; rather, we aimed to provide a structured synthesis of the available evidence. Searches were conducted up to July 2025. We graded the strength of the evidence identified according to the following criteria employed in similar narrative and systematic reviews [28, 29, 30]: (1) inconclusive evidence: 1–2 studies available, with inconsistent or conflicting results; (2) weak evidence in favor: 3–5 studies supporting the association, with fewer than 3 reporting negative or null findings; (3) strong evidence: more than 5 studies supporting the association and/or at least one systematic review or meta‐analysis, with few negative or null results; and (4) no evidence: no identified studies examining the association.

## Summary of the evidence

**Fig. 1:**
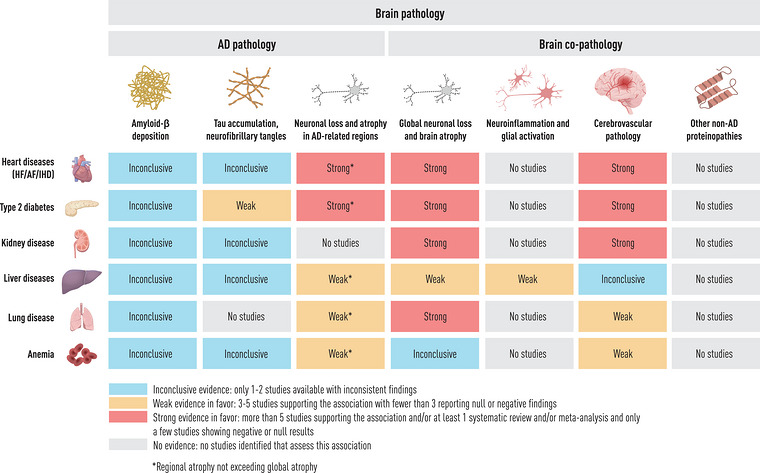
Overview of associations between somatic conditions and brain pathologies. For the complete overview of studies investigating each association refer to the main text and Table 1.

Across the somatic diseases examined, the strongest and most consistent evidence emerged for associations with diffuse neuronal loss or global brain atrophy and cerebrovascular pathology (Fig. 1 and Table 1).

**Table 1:** Complete overview of studies investigating associations between somatic conditions and brain pathologies included in this narrative review.

	Brain pathology													
	AD pathology						Brain co‐pathology							
	Amyloid‐β deposition		Tau accumulation, neurofibrillary tangles		Neuronal loss and atrophy in AD‐related regions		Global neuronal loss and brain atrophy		Neuroinflammation and glial activation		Cerebrovascular pathology		Other non‐AD proteinopathies	
	Association	No association	Association	No association	Association	No association	Association	No association	Association	No association	Association	No association	Association	No association
**Heart diseases (HF/AF/IHD)**	(Beeri, 2006)	(Dublin, 2014; Ihara & Washida, 2018; Johansen, 2022; Sposato, 2017; Starmans, 2023; Vemuri, 2017)	(Beeri, 2006)	(Dublin, 2014; Ihara & Washida, 2018; Sposato, 2017; Vemuri, 2017)	(Alosco & Hayes, 2015; Carbone, 2024; Frey, 2018; Knecht, 2008; Koschack & Irle, 2005; Moazzami, 2020; Schwennesen, 2024; R. L. C. Vogels, 2007; Yaqub, 2025; Yun, 2022)	(Graff‐Radford, 2016)	(Almeida, 2008, 2012; Alosco & Hayes, 2015; Barekatain, 2014; Berman, 2019; Carbone, 2024; Frey, 2018; Graff‐Radford, 2016; Liu, 2025; Lyra, 2020; Moazzami, 2020; Ottens, 2017; Piers, 2016; Stefansdottir, 2013; Vemuri, 2017; R. L. C. Vogels, 2007; W. Wang, 2025; Yaqub, 2025; Yun, 2022; Zheng, 2023)	(Almeida, 2013; Knecht, 2008)	–	–	(Alosco, Brickman, Spitznagel, Garcia, 2013; Alosco, Brickman, Spitznagel, Griffith, 2013; Berman, 2019; Carbone, 2024; de Leeuw, 2000; Ding, 2021; Dublin, 2014; Fanning, 2014; Ferro, 2020; Frey, 2018; Graff‐Radford, 2016; Koh, 2022; T. Li, 2023; Rydén, 2021; Sposato, 2017; Stegmann, 2020; R. L. C. Vogels, 2007; W. Wang, 2025)	–	–	–
**Type 2 diabetes**	(McIntosh & Nation, 2019; van Arendonk, 2022)	(Luchsinger, 2020; Moran, 2015; Pruzin, 2018; Roberts, 2014; Van Gils, 2024)	(Moran, 2015; Van Gils, 2024)	(Ennis, 2023; Pruzin, 2018)	(Motaghi, 2024; T. Zhang, 2022)	–	(Motaghi, 2024; T. Zhang, 2022)	–	–	–	(Biessels, 2020; Pruzin, 2018)	–	–	–
**Kidney disease**	(Sun, 2023)	(Dittrich, 2023)	(Sun, 2023)	(Dittrich, 2023)	–	–	(Chang, 2017; Dong, 2018; Song, 2008; S. C. M. Vogels, 2012; Yeh, 2019; L. J. Zhang, 2013)	(Dittrich, 2023)	–	–	(Fanning, 2014; Khatri, 2007; Kobayashi, 2009; Vinters, 2021; S. C. M. Vogels, 2012; Yeh, 2019)	–	–	–
**Liver diseases**	(Kang, 2023; Weinstein, 2022)	(Weinstein, 2022)	(Weinstein, 2022)	(Weinstein, 2022)	(Leone, 2023; Lu, 2024; Parikh, 2023; Weinstein, 2022)	(Weinstein, 2018, 2024)	(Parikh, 2023; Weinstein, 2018, 2024)	–	(Balzano, 2018; Cagnin, 2006; Leone, 2023)	(Zemtsova, 2011)	(Balzano, 2018; Lu, 2024)	(Parikh, 2023; Weinstein, 2024)	–	–
**Lung disease**	(Nair, 2023)	–	–	–	(Carlson, 2017; Han & Zhao, 2025; J. Li & Fei, 2013)	–	(Chen, 2016; Dodd, 2012; Savage, 2018; Spilling, 2019; C. Wang, 2017; Yin, 2019; H. Zhang, 2012, 2013)	(Borson, 2008; Ryu, 2013)	–	–	(Lahousse, 2013; van Dijk, 2004; Xiao, 2022)	(van Dijk, 2004)	–	–
**Anemia**	(Yang, 2021)	(Kim, 2021)	–	(Kim, 2021; Yang, 2021)	(Beydoun, 2021; Kim, 2021; Omori, 2024)	–	(Omori, 2024; Qiang, 2023)	–	–	–	(Beydoun, 2021; Van Der Veen, 2015; Wolters, 2019)	(Kim, 2021)	–	–

Abbreviations: AF, atrial fibrillation; HF, heart failure; IHD, ischemic heart disease.

### Neuronal loss and brain atrophy

Strong and consistent evidence indicates diffuse neuronal loss and global brain atrophy across most examined somatic conditions [19, 31, 32, 33, 34, 35, 36, 37, 38, 39, 40, 41, 42, 43, 44, 45, 46, 47, 48, 49, 50, 51, 52, 53, 54, 55, 56, 57, 58, 59, 60, 61, 62, 63, 64, 65], with the exception of (1) liver disease [66, 67, 68], for which data remain limited, and (2) anemia [69, 70], where findings were inconclusive. Although medial temporal lobe and hippocampal atrophy have been reported to be associated with several somatic chronic conditions [19, 31, 32, 47, 48, 49, 53, 56, 57, 66, 70, 71, 72, 73, 74, 75, 76, 77, 78, 79, 80, 81], these regional changes typically do not exceed global brain atrophy. For chronic kidney disease, no conclusions can be drawn regarding AD‐specific regional atrophy due to the lack of studies. Overall, the pattern of brain atrophy observed for most diseases suggests that neuronal loss related to somatic diseases is rather diffuse and not confined to AD‐specific regions, indicating a generalized rather than selective neuropathological process.

### Cerebrovascular pathology

Strong and consistent evidence indicates that cerebrovascular pathology is present across most examined somatic conditions, with the exception of liver disease, for which data remain limited [66, 67, 68, 78, 82]. Converging findings from large imaging and autopsy studies show robust associations between most somatic diseases and markers of vascular brain injury, including white matter lesions, lacunes, and large infarcts [17, 18, 29, 41, 44, 48, 49, 56, 60, 61, 62, 77, 83, 84, 85, 86, 87, 88, 89, 90, 91, 92, 93, 94, 95, 96, 97, 98, 99, 100, 101], possibly reflecting both small and large vessel disease processes. Importantly, these associations are observed across different study designs and measurement approaches, supporting the consistency of the evidence. Taken together, these findings support the interpretation that cerebrovascular damage may represent a pathway linking somatic diseases to brain pathology.

### Amyloid‐β deposition, tau accumulation, and neurofibrillary tangles

Evidence on the relationship between somatic diseases and AD‐related pathology—specifically amyloid and tau accumulation—remains limited and inconsistent. Although some studies, particularly in the context of untreated type 2 diabetes [85], have reported associations with increased amyloid PET burden [102] or altered CSF tau/Aβ42 ratios [103], others have failed to confirm these findings, even in large and well‐characterized cohorts [104, 105, 106, 107]. For example, type 2 diabetes has been associated with elevated tau biomarkers but not with Aβ biomarkers in a recent systematic review and meta‐analysis conducted by van Gils et al. [108]. An association between anemia and CSF amyloid, but not tau, levels was also reported [109], whereas Kim et al. found no association with either amyloid or tau PET scans [81]. Similarly, a small study in individuals with chronic kidney disease suggested possible alterations in CSF amyloid and tau levels [110], but these results have not been replicated in a larger population‐based sample [111]. Evidence for liver disease is heterogeneous. A female‐specific relationship between nonalcoholic fatty liver disease (NAFLD) and Aβ deposition was described [112], although prevalent NAFLD was not associated with Aβ and tau PET deposition for either sex among Framingham Study participants [80]. Among the different conditions examined, heart diseases, and in particular atrial fibrillation and heart failure, have been investigated more extensively. For instance, chronic cerebral hypoperfusion derived from persistent atrial fibrillation may promote amyloid and tau pathology by increasing Aβ production, reducing its clearance, and enhancing tau phosphorylation [113]. Similarly, an autopsy study found that coronary artery disease was associated with increased densities of neuritic plaques and neurofibrillary tangles [21]. Nonetheless, current evidence remains mixed [113] and does not support associations between heart diseases and amyloid or tau pathology, as shown by several imaging [55, 114] and neuropathological [83, 96] studies, including a recent systematic review and meta‐analysis [20]. Indeed, the authors of the latter found no evidence supporting an association between atrial fibrillation, alone or in combination, with other cardiac diseases and increased cerebral Aβ burden, suggesting that concomitant vascular brain injury may underlie the association between heart disease and the clinical diagnosis of dementia [20]. Finally, one recent meta‐analysis found an increased amyloid burden among patients with obstructive sleep apnea [115], although future studies are needed to address the impact of lung disorders in AD pathology [116]. Overall, current evidence that links somatic diseases with amyloid or tau pathology remains limited.

### Neuroinflammation and non‐AD proteinopathies

We have identified no studies that systematically examined the presence of chronic somatic diseases in relation to neuroinflammation or non‐AD proteinopathies (e.g., α‐synuclein and TDP‐43). Limited evidence is available for liver disease, particularly in the context of cirrhosis and steatohepatitis [79, 82, 117]. In fact, patients with steatohepatitis or liver cirrhosis showed altered microglial phenotypes, immune cell infiltration, apoptosis and neuronal loss in one prior study [79], and cerebellar neuroinflammation seemed to occur at early stages of liver disease [82]. However, findings remain heterogeneous and often conflicting, as microglia exhibited a context‐dependent response profile, with evidence of phenotypic changes primarily observed in the presence of hepatic encephalopathy [118].

Overall, the available evidence is insufficient to establish a clear association between somatic diseases and neuroinflammatory processes or non‐AD proteinopathies.

## Results interpretation and unifying framework

Based on the findings of this narrative review, three main conclusions can be drawn. First, chronic somatic diseases were consistently associated with diffuse neuronal loss and global brain atrophy, extending beyond regions typically vulnerable to AD [19, 31, 32, 33, 34, 35, 36, 37, 38, 39, 40, 41, 42, 43, 44, 45, 46, 47, 48, 49, 50, 51, 52, 53, 54, 55, 56, 57, 58, 59, 60, 61, 62, 63, 64, 65, 66, 67, 68, 69, 70]. Second, there was robust and consistent evidence linking these conditions to cerebrovascular pathology, including white matter lesions, infarcts, and microvascular damage [17, 18, 29, 41, 44, 48, 49, 56, 60, 61, 62, 77, 78, 82, 83, 84, 85, 86, 87, 88, 89, 90, 91, 92, 93, 94, 95, 96, 97, 98, 99, 100, 101]. Third, evidence remains limited and inconclusive regarding their association with the core pathological hallmarks of AD, namely, amyloid and tau accumulation.

Taken together, these findings suggest that somatic disease burden may contribute to brain pathology through mechanisms that are (at least partly) independent of classical AD‐related processes. Although this review focused on neuropathological rather than clinical endpoints, the conditions examined were selected because they are both highly prevalent in aging populations and well‐established risk factors for dementia [6, 7, 8, 9]. These observations support a plausible, albeit still speculative, mechanistic interpretation: Somatic diseases likely contribute to dementia development primarily through non‐AD pathways, particularly cerebrovascular injury and diffuse neurodegeneration, rather than through direct acceleration of amyloid or tau pathology.

The conditions examined in this review are highly prevalent in aging populations, implying that mixed brain pathology is likely the rule rather than the exception in their presence. This reinforces the concept of a body–brain connection in dementia development in late life [6]. Notably, cerebrovascular damage and diffuse neuronal loss emerged as recurrent features across most of the diseases examined, suggesting that both processes may represent key pathways linking systemic somatic burden to central nervous system injury.

Importantly, this framework does not exclude a role of AD pathology. Rather, even if somatic diseases do not directly drive AD‐specific pathology, the brain alterations they induce could lower the threshold at which existing AD pathology becomes clinically manifest, thereby influencing the clinical expression of dementia [119].

In this context, systemic disease burden may shift the balance from compensated to decompensated brain function [120], accelerating the transition from preclinical or prodromal to clinical stages of cognitive impairment without necessarily driving the core molecular features of AD itself.

This interpretation has important implications for clinical practice. It underscores the need to move beyond a brain‐centric approach to dementia [121] and reinforces the concept of “mixed dementia” as a central feature of cognitive impairment in late life. Rather than reflecting a single pathogenic process, dementia in older adults likely emerges from the cumulative and interacting effects of multiple neuropathological and systemic conditions [27, 122, 123, 124]. This perspective supports a comprehensive approach to older adults [125, 126, 127, 128], in which the prevention and treatment of somatic diseases are considered central components of dementia risk reduction. Targeting cardiovascular, metabolic, and systemic health may yield substantial benefits not only by reducing vascular brain injury but also by preserving overall brain resilience [129, 130].

## Future perspectives

The findings of this review highlight several critical gaps in the literature and define key priorities for future research.

The relationship between somatic diseases and the core pathological hallmarks of AD remains poorly characterized, with existing evidence being inconsistent and heterogeneous. Several factors may account for these inconclusive findings. First, although targeted searches were designed to maximize sensitivity, the present review was not systematic, and some relevant studies may therefore have been missed, particularly those in which neuropathological alterations were reported as secondary outcomes. However, this limitation is unlikely to fully explain the limited amount of available evidence identified in the literature. Further, this review was restricted to studies conducted in humans, thus excluding experimental work in animal models that could provide mechanistic insights into how somatic diseases influence AD‐related processes. Although this restriction may limit the overall coverage of our synthesis, it underscores the need for longitudinal human studies integrating multimodal biomarkers to clarify whether chronic somatic conditions directly initiate or accelerate amyloid and tau pathology, or instead primarily contribute to non‐AD forms of brain injury and modify the clinical expression of underlying neuropathology [131].

Another major gap in the literature is the limited evidence on the association between somatic diseases and neuroinflammation. Although neuroinflammation is a central mechanism in aging and in the pathogenesis and progression of dementia [132], it remains unclear whether and to what extent systemic health directly influences brain inflammatory processes in humans or if inflammation is a consequence of initial and more pathognomonic pathological processes. Likewise, no studies have systematically examined the association between somatic conditions and non‐AD proteinopathies, such as α‐synuclein or TDP‐43, despite their high prevalence and their established role in cognitive decline [27]. Addressing these gaps will be fundamental to advancing our understanding of how systemic health influences the full spectrum of neuropathological changes underlying dementia.

This review focused on postmortem data, neuroimaging (MRI and PET), and CSF‐based biomarkers, which have been the most widely implemented tools in research and clinical practice. However, blood‐based biomarkers have recently emerged as promising alternatives, showing strong correlation with AD neuropathology and CSF measures [133], and accurately predicting dementia in both clinical [134] and community‐based [10, 135, 136] settings. Importantly, growing evidence indicates that several somatic diseases—including those examined in this review—are associated with altered circulating concentration of these molecules in the blood [119, 136, 137, 138, 139, 140]. This highlights the need to further explore whether such alterations reflect underlying pathophysiological processes (such as a synergistic contribution to the clinical expression of dementia) or arise independently of brain pathology (e.g., via peripheral mechanisms). Future studies should clarify whether disease‐related alterations in blood biomarkers reflect underlying brain pathology, peripheral biological processes, or a combination of both. This will be essential for interpreting biomarker profiles in older adults with multimorbidity and for advancing a more precise understanding of the body–brain crosstalk in dementia development.

Finally, future research should address sex‐specific differences in the relationship between somatic diseases and brain pathology. Many somatic conditions [141, 142, 143] and dementia‐related neuropathologies [144, 145, 146] clearly show sex differences in biological and clinical expressions, yet studies specifically addressing sex differences are sparse and inconsistent. Understanding whether systemic diseases differentially affect brain vulnerability in women and men may help explain heterogeneity in dementia trajectories and enable more accurate risk stratification and personalized prevention strategies in late life.

## Clinical and public health implications

The interpretation of the results proposed in this review has important clinical implications, informing both therapeutic approaches and preventive strategies for dementia in late life.

The traditional view of AD as a disorder driven by the accumulation of a single misfolded protein has shaped decades of research. In this context, recent therapeutic advances—such as the FDA and EMA approval of lecanemab and donanemab, anti‐amyloid monoclonal antibodies—represent important milestones [147, 148]. However, data suggest that only a minority of patients with mild or early AD dementia would be eligible for such treatment [149, 150], due to either the absence of isolated amyloid pathology or the presence of significant comorbidities [151, 152]. This mismatch between trial populations and real‐world patients [153] underscores the need for a broader and more nuanced understanding of dementia biology, accounting for the high prevalence of brain co‐pathologies and systemic health conditions in aging populations [6, 24, 154, 155]. Importantly, although mixed neuropathological profiles are increasingly recognized as a hallmark of late‐life dementia, a formal definition of “mixed dementia” is still lacking. Advancing toward a biologically informed and clinically meaningful definition of “mixed dementia” will be essential to improve patient characterization and develop personalized therapeutic approaches.

Moving beyond a single‐pathway model is therefore essential to delivering personalized care, by combining strategies that also target non‐AD‐related pathologies and coexisting morbidities [131, 154, 156]. To this end, future research should also focus on identifying panels of biomarkers capable of capturing the clinical and biological heterogeneity of late‐life dementia, as well as its interplay with somatic health. Such an approach will be essential to translating recent therapeutic advances into meaningful benefits for older adults with heterogenous clinical profiles. Moving forward, integrating systemic health assessments with advanced biomarker profiling will enable a deeper understanding of dementia pathophysiology and support the development of personalized therapeutic strategies for older adults with complex health profiles.

Importantly, findings of this review also have implications for dementia prevention. If chronic somatic diseases primarily act by accelerating diffuse neuronal loss and cerebrovascular damage, rather than directly inducing AD pathology, then preventing, delaying, or optimally managing these conditions will represent a key strategy to preserve brain resilience in late life. This perspective reinforces the potential of multimodal preventive approaches as a cornerstone of dementia prevention, where the combination of optimal management of cardiovascular and metabolic risk factors with lifestyle interventions—including physical activity, nutritional optimization, and smoking cessation—can mitigate accelerated brain aging and may ultimately reduce dementia risk and delay dementia onset [121, 157, 158]. Such approaches will likely be central to both the prevention and treatment of dementia in late life, where mixed brain pathology is common and complex clinical profiles are frequently observed in very old people.

## Conclusion

In conclusion, this review highlights how systemic health shapes brain integrity in aging, with consistent evidence linking chronic somatic diseases to diffuse neuronal loss and cerebrovascular injury but limited and largely inconclusive associations with AD‐specific pathology. Taken together, these findings support a model in which somatic diseases do not primarily act as direct drivers of amyloid or tau accumulation, but rather as modifiers that lower brain resilience and accelerate the clinical expression of underlying neuropathology.

This body–brain perspective emphasizes the need to move beyond a purely brain‐centric approach and reinforces the concept of “mixed dementia” as a central feature in old age. Advancing this view will be essential to improve risk stratification, inform prevention strategies, and guide the development of more inclusive and clinically relevant therapeutic approaches for aging populations characterized by complex health profiles.

## Author contributions

G. Grande and M. M. Mielke contributed to the conceptualization and design of the review, supervised the work, and drafted the figure. M. Valletta, F. Gasparini, and J. R. Bacci performed the main literature search and contributed to study selection and evaluation. G. Grande drafted the manuscript and developed the initial outline. All authors critically revised the manuscript, provided intellectual input, and approved the final version.

## Conflict of interest statement

The authors declare no conflicts of interest.

## Funding information

GG received financial support from the Karolinska Institutet Strategic Research Area in Neuroscience in 2025 and the Karolinska Institutet Committee for Research in 2025. FG received financial support from Stiftelsen Dementia. DLV received financial support from the Karolinska Institutet Strategic Research Area in Epidemiology and Biostatistics in 2021 and 2023, the Karolinska Institutet Strategic Research Area in Neuroscience in 2025 and the Karolinska Institutet Committee for Research in 2026.

## Data Availability

The data that support the findings of this study are available on request from the corresponding author. The data are not publicly available due to privacy or ethical restrictions.
